# A Comment on the November Issue of POCUS Journal

**DOI:** 10.24908/pocus.v7i2.16034

**Published:** 2022-04-21

**Authors:** Benjamin T Galen

**Affiliations:** 1 Editor-in-Chief, POCUS Journal

**Keywords:** POCUS

Dear readers,

This is a very exciting issue of POCUS Journal.  We have two new sections: a book review and Innovations in POCUS Curriculum.  While there are many online and electronic resources for POCUS, textbooks still have an important, but evolving role in medical education. We appreciate the opportunity to review new POCUS textbooks in POCUS Journal. Please see page 184 as our first book review.

Another new peer-reviewed article type is “Innovations in POCUS Curriculum”. We realized over the years that many innovative POCUS educational and training curricula are not always able to be evaluated with rigorous study outcomes. We believe that the evolution of POCUS training across the spectrum from students to graduate medical trainees to the continuing medical education setting requires the sharing of ideas. We hope that providing a forum to share POCUS curricula that are not necessarily validated by educational research methods permits ongoing collaboration between educators as well as ongoing local support for curriculum development. For this first issue see page 185 by Nauka et al.

Lastly, with this November 2022 issue there emerged a theme in the case files and case reports: soft tissue examination using POCUS. Sheeka, et al. describe a diagnostic radiology case of a retained foreign body, but this is very important for POCUS users to learn from. Kaiyasah, et al. describe POCUS application in pilonidal sinus disease, a novel application for this common disease entity. Freilich et al. present a case of a soft tissue mass that was highly vascularized on POCUS examination, surprisingly found to be a post-traumatic AVM. McCreary, et al. describe the POCUS findings in a young patient with extraosseous Ewing’s sarcoma in the neck. Taken together, these cases highlight how useful POCUS is in soft tissue examination beyond basic indications such as evaluating cellulitis for underlying abscess. While some of the above entities might be “rare”, POCUS users who scan soft tissue should be aware of these cases and use the discussion to expand their differential diagnosis of soft tissue findings accordingly. 

Sincerely,

Benjamin T. Galen, MD

Editor-In-Chief 

POCUS Journal

